# Intercepting the NF1 course through early MEK inhibition

**DOI:** 10.1016/j.xinn.2026.101597

**Published:** 2026-09-30

**Authors:** Nicolas André, Laura Fertitta, Pierre Leblond, Mona Amini-Adle, Pierre Wolkenstein

**Affiliations:** 1 Pediatric Hematology and Oncology Department, Hôpital pour Enfant de la Timone, AP-HM, 13005 Marseille, France; 2 REMAP4KIDS, Centre de Recherche en Cancérologie de Marseille (CRCM), Aix-Marseille Université, CNRS, INSERM, 13273 Marseille, France; 3 Metronomics Global Health Initiative, 13005 Marseille, France; 4 Mondor Institute for Biomedical Research (IMRB), INSERM, Créteil, France; 5 Department of Dermatology, Reference Center for Neurofibromatosis, Henri-Mondor Hospital, AP-HP, 94000 Créteil, France; 6 Université Paris Est Créteil, UPEC, 94000 Créteil, France; 7 Institute of Pediatric Hematology and Oncology (IHOPe), Centre Léon Bérard, 69008 Lyon, France; 8 Department of Dermatology, Centre Léon Bérard, 69008 Lyon, France

## Main text

Recent clinical trials led to the regulatory approval of two MEK inhibitors (MEKis), selumetinib^1^ and mirdametinib, for the treatment of plexiform neurofibroma (PN) in both pediatric and adult patients with type 1 neurofibromatosis (NF1). While treatment with MEKis represents a genuine breakthrough in NF1 offering the first targeted therapies for patients, clinicians and patients and their families currently face a frustrating paradox. Indeed, up-to-date indications focus on treating inoperable, symptomatic PN, but preclinical scientific and clinical data are emerging for a broader clinical impact of MEKi treatment on NF1 manifestations.^1^^,^^2^^,^^3^

We advocate for an early treatment to intercept the disease course and harness the potential of MEKis not only to mitigate tumor burden but also, for instance, to potentially improve long-term outcomes in cognition, so that the therapeutic clinical impact could be much wider than currently reported.

## Precedents for early intervention for orphan disease

This strategy has already been gradually used, for instance, for tuberous sclerosis complex with mtor inhibitors. Initially, everolimus was reported for the treatment of subependymal giant cell astrocytomas but now represents a disease-modifying treatment for other aspects of tuberous sclerosis, such as epilepsy, where a 40% decrease in resistant focal onset seizures was observed, and renal (angiomyolipoma) and cardiac manifestations. Elsewhere, the serendipitous improvement of neurological and cardiological function in children with D-2-hydroxyglutaric aciduria type II^4^ upon treatment of their IDH2-mutated midline glioma with enasidenib (an IDH2 inhibitor) led to the initiation of treatment with enasidenib before the occurrence of any diffuse midline glioma, confirming that early treatment could lead to a significant decrease of symptoms.^4^

## The cognitive window of opportunity

Cognitive impairment, including executive dysfunction and attention-deficit/hyperactivity disorder, affects up to 80% of NF1 patients and contributes to lifelong morbidity.^1^^,^^3^ RAS hyperactivation and subsequent hyperactivation of the RAS/ERK pathway negatively affects neuroplasticity and memory. Clinical data have shown preliminary and promising neurocognitive benefits in 59 patients ages 5–27 years treated with MEKis for 48 weeks, who experienced significant improvements in executive functions.^5^ Elsewhere, postive effects of trametinib on congition in 36 pediatric patients have been reported with substantial improvements in processing speed and visuomotor and verbal habilities.^5^ Initiating MEKis earlier may lead to normalization of RAS/ERK signaling before permanent structural or functional neurocognitive deficits become fixed, maximizing the opportunity for cognitive protection/improvement.

## Closing the gap on plexiform neurofibroma growth

PNs are a hallmark of NF1; they are often present by the age of 5 and grow faster in children.^2^ PNs represent a source of chronic morbidity with pain and disfigurement. Of note, the KOMET trial in 145 adult patients^3^ reported lower response rates to selumetinib (20% ORR [overall response rate]) than the SPRINT trial in 50 children^1^ (68% ORR). A smaller difference was observed between adults and children in the ReNeu trial evaluating mirdametinib (51% vs. 42% (NCTT 03962543). This suggests that, as patients age, their PNs become more fibrotic and less responsive to therapy. By waiting for symptoms to become “severe enough,” we might essentially be waiting for the treatment to become less effective. Thus, the current “watch-and-wait” approach risks missing the window for optimal medical intervention with MEKis. Early preemptive treatment could halt PN growth before they become inoperable or symptomatic. Furthermore, if initiated earlier, treatment would need only to stop PN growth instead of reducing the tumors to bring benefit to the patients by preventing associated long-term complications. This is the aim of the NCT06188741 ongoing trial. It shall enroll 200 patients with asymptomatic PNs in high-risk locations to determine if participants benefit from early selumetinib treatment. We aim to go a step further by initiating treatment even in asymptomatic patients and by focusing not only on PNs but on other symptoms of the disease.

## Preventing malignant transformation?

The most significant cause of mortality in NF1 patients is the development of malignant peripheral nerve sheath tumors (MPNSTs), which arise predominantly from preexisting PNs, have a lifetime risk ranging from 8% to 15%.^2^ The transformation from benign PN to MPNST involves the accumulation of additional genetic mutations on a background of RAS hyperactivation. Anecdotal case reports have described MPNSTs in patients receiving MEKis, so that the potential of MEKis to reduce the risk of malignant transformation remains an open question. Nevertheless, the ability of MEKis to shrink PNs and limit their growth if started earlier suggests that early use might reduce the overall tumor burden and, theoretically, the pool of cells susceptible to malignant transformation, thereby lowering the lifelong risk of MPNSTs. This hypothesis requires prospective evaluation, but given the dismal prognosis of MPNST, the potential preventative benefit warrants serious consideration in treatment strategy especially in patients with risk factors. Furthermore, early intervention may also influence the progression of other NF1-associated tumors. Indeed, MEKis can also shrink or stabilize frequent NF1-associated optic pathway gliomas (OPGs), and early treatment could prevent definitive vision alterations instead of limiting them once present.

## A call to action: Ready to MINGLE?

The evidence supporting early initiation of MEKis in NF1 children is compelling, stemming from strong molecular rationale and promising clinical signals. Shifting our therapeutic strategy from “wait and see” to “early and proactive” holds the potential to correct the natural history of children with NF1 through, for instance, improving cognitive outcomes, reducing lifelong morbidity, and potentially lowering the risk of malignant transformation.

To test this hypothesis, we propose the MINGLE approach: MEK Inhibition for Neurofibromatosis 1 Global, Lifelong Evaluation. Under this framework, we advocate for the administration of MEKis within a clinical trial setting (Figure 1).

**Figure 1:**
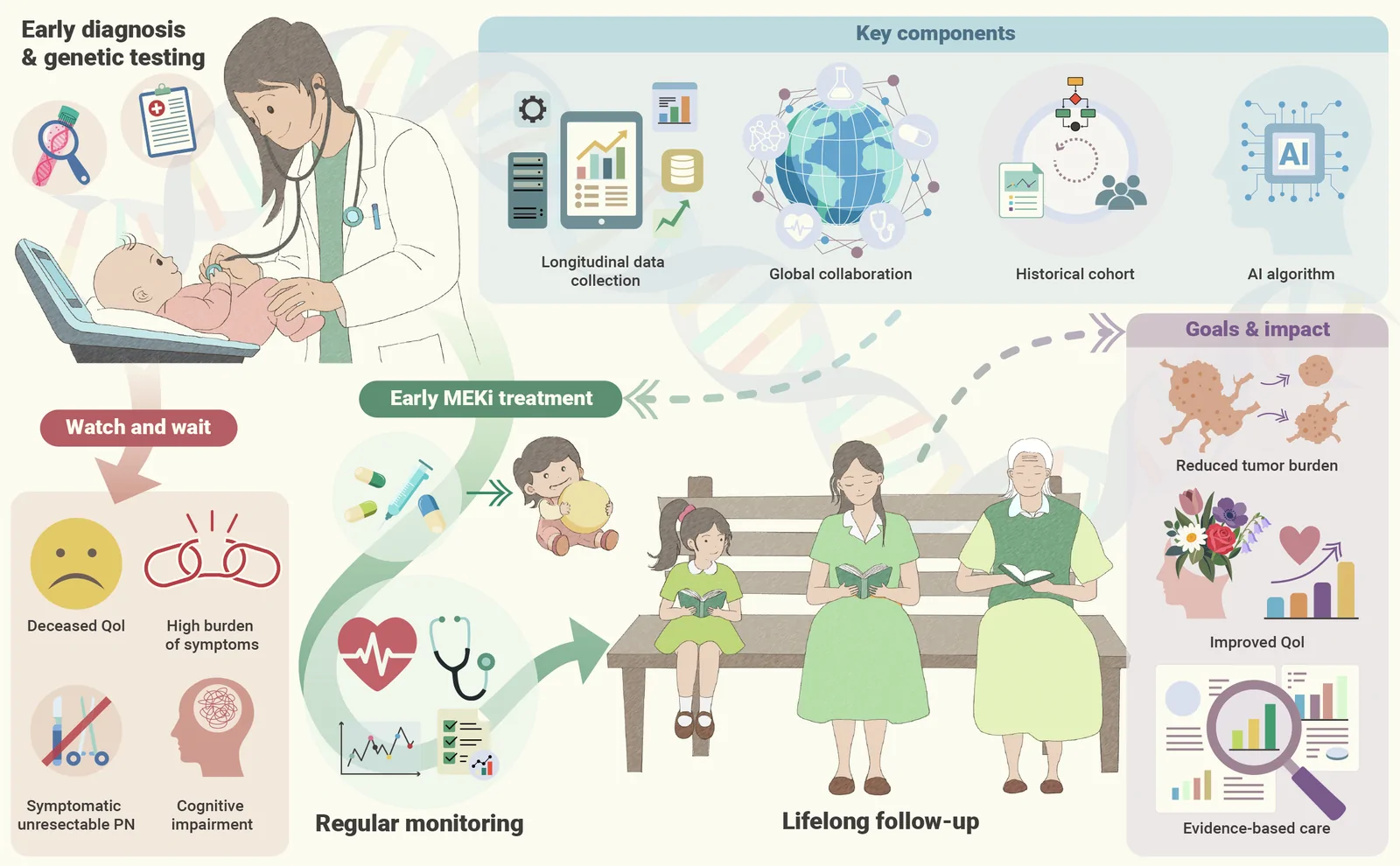
The MINGLE process MINGLE aims at starting an early treatment with MEK inhibitors for NF1 patients before the burden of symptoms is high. The duration of treatment will be based on toxicity/benefit balance and will be monitored during the patients’ whole life. This international project will aim at improving quality of life, reducing the symptoms through AI analysis to individualized treatment evaluation and recommendations.

Bringing early MEKi treatment to standard of care is not an easy task and presents major clinical, methodological, and structural challenges.

We plan to enroll asymptomatic/paucisymptomatic patients who meet international diagnostic criteria, such as children with a confirmed molecular diagnosis and affected parents. Alternatively, an adaptive risk-based approach will be used (e.g., NF1 microdeletions, codon 844–848 mutations, or high-risk PN/OPG locations). Standard criteria for initiating MEKi therapy must also be met. To optimize study design in the context of a rare disease, the trial would also integrate an *in silico* control arm based on historical cohorts as a comparator. These data will feed and be processed by an artificial intelligence (AI) algorithm designed to support clinical decision-making for individual patients.

The primary hurdle remains the delicate calibration of the benefit-risk ratio, where the potential for chronic, long-term exposure to known class-effect toxicities must be meticulously weighed against the slow progression of benign tumors, the multiple clinical symptoms/manifestations, and the anticipated potential preventive clinical benefits. In cases of grade 3/4 toxicities, to improve tolerance/compliance, dose reduction or alternative dose schedules will be proposed to patients. The assessment of efficacy is complicated by the requirement for long-term follow-up, the necessity of longitudinal monitoring of multiple heterogeneous endpoints, where primary and secondary endpoints need to be individualized and could change over time even for a given patient (i.e., OPG until 18 years old and PN at older age). The potential for adaptive resistance or compensatory signaling pathways that may emerge during the course of prolonged therapeutic intervention also needs to be taken into account. We would ensure a longitudinal follow-up in which, in addition to standard imaging follow-up, repeated neurocognitive evaluations and patient-reported outcomes would be central to evaluate toxicity and efficacy.

Furthermore, the trial design must take into account the substantial clinical and genetic heterogeneity of the NF1 population, which contributes to a highly variable natural history. Last, while the integration of an *in silico* cohort can effectively address recruitment limitations inherent to rare disease populations, it necessitates careful calibration to mitigate the risks of covariate imbalance and selection bias, which may arise if external clinical datasets lack rigorous, standardized longitudinal collection.

The MINGLE project will also require a robust, integrated framework of organization, funding, and governance. MINGLE will operate as a federated international consortium, uniting academic clinical centers, NF1 advocacy groups, pharmaceutical companies, and regulatory stakeholders. A sustainable funding strategy will center on public-private partnerships, leveraging joint investments from pharmaceutical stakeholders and public health agencies to support the transition of MEKis from research to standard of care. Core funding will be secured through a combination of philanthropy, research grants, and direct industry contributions. To ensure effective oversight, a steering committee composed of key stakeholders will supervise all critical project aspects, including protocol development, AI-driven evidence generation, clinical trial execution, and data quality assurance.

Thus, overall, while we must, of course, proceed with caution regarding patient selection, long-term safety and optimal dosing, and duration of treatment, the cost of inaction is currently too high. We need a concerted effort to launch prospective trials that explore this new frontier. It is time to stop managing the damages of NF1 and start actively preventing them. We owe it to our patients to turn these clinical signals of activity into a new standard of care that prioritizes early interception and unveils other potential benefits of early treatment. So, ready to MINGLE?

## Declaration of interests

N.A. has had an advisory role (Alexion, IPSEN, and Norgine) and has received grants through his institution from Bristol Myers Squibb and drugs for trials from Bristol Myers Squibb, Pierre Fabre, Pfizer, and AstraZeneca and travel support from Roche, Novartis, Alexion, and IPSEN; he further has independent data monitoring committee roles for Accord Healthcare and ITM. P.W. has had an advisory role for and travel support from Alexion. P.L. has had advisory roles for and travel support from Alexion, Novartis, and IPSEN.
